# 

**Published:** 1865-08

**Authors:** 


### CONTENTS.

ORIGINAL ESSAYS AND COMMUNICATIONS.

Dr. Brainard's Address before the American Dental Association ........ 303
Thermal Effects of Dental Filling in Teeth ........ 317
An Address of Welcome to the American Dental Association, at Chicago ........ 324
On the Use of Chloroform and Ether in Dental Operations ........ 332
Pleasures and Perplexities of Dental Practice ........ 338
Deciduous Teeth; Their Importance; Their Preservation ........ 342
A Call to Organize a Society ........ 345

EDITORIAL.

Medical Specialties ........ 346
The American Dental Association ........ 346
Central Ohio Dental Society ........ 347
The American Dental Convention ........ 348

